# Effect of temperature on the carbonization process of cationic carbon dots: a physicochemical and *in vitro* study†

**DOI:** 10.1039/d5ra00062a

**Published:** 2025-04-28

**Authors:** Nicolás Santos, Paula A. Santana, Igor Osorio-Roman, Carlos Jara-Gutiérrez, Joan Villena, Manuel Ahumada

**Affiliations:** a Centro de Nanotecnología Aplicada, Facultad de Ciencias, Ingeniería y Tecnología, Universidad Mayor Camino La Pirámide 5750, Huechuraba Santiago RM Chile Manuel.ahumada@umayor.cl; b Instituto de Ciencias Aplicadas, Facultad de Ingeniería, Universidad Autónoma de Chile El Llano Subercaseaux 2801 Santiago San Miguel Chile; c Instituto de Ciencias Químicas, Facultad de Ciencias, Universidad Austral de Chile Isla Teja s/n Valdivia Región de los Ríos Chile; d Centro Interdisciplinario de Investigación Biomédica e Ingeniería para la salud (MEDING), Escuela de Kinesiología, Facultad de Medicina, Universidad de Valparaíso Valparaíso Chile; e Centro Interdisciplinario de Investigación Biomédica e Ingeniería para la salud (MEDING), Escuela de Medicina, Facultad de Medicina, Universidad de Valparaíso Valparaíso Chile; f Escuela de Biotecnología, Facultad de Ciencias, Ingeniería y Tecnología, Universidad Mayor Camino La Pirámide 5750, Huechuraba Santiago RM Chile

## Abstract

This work highlights the critical role of synthesis conditions in tuning the properties of carbon dots (CDs) for optimized performance in biomedical applications, offering valuable insights into the design of these carbon nanomaterials. Although various synthesis methods and carbon sources have been explored for CD production, few studies have investigated how synthesis temperature modulates and optimizes their physicochemical attributes. In this study, cationic CDs derived from poly(ethylene imine) (PEI) and chitosan (CS) were synthesized using a microwave-assisted hydrothermal method at different temperatures to explore this aspect. It was found that higher carbonization temperatures during the hydrothermal process resulted in smaller, more photoluminescent CDs. This increase in temperature significantly enhanced the biological interactions of the CDs, demonstrating notable biocompatibility. In contrast, the lowest hydrothermal temperature enhanced cytotoxic effects against the Gram-positive pathogen *Staphylococcus aureus* under light exposure. Furthermore, gastric cancer (AGS), colon cancer (HT-29), cervical cancer (HeLa), prostate cancer (PC-3), and breast epithelial (MCF-10) cell lines showed cytotoxicity that was dependent on the CDs synthesized at different temperatures.

## Abstract 2

Carbon dots prepared at different carbonization temperatures showed an increase in their optical properties, along with a decrease in cytotoxicity towards bacterial strains and both tumor and non-tumor cell lines as the temperature increased.

## Graphical abstract

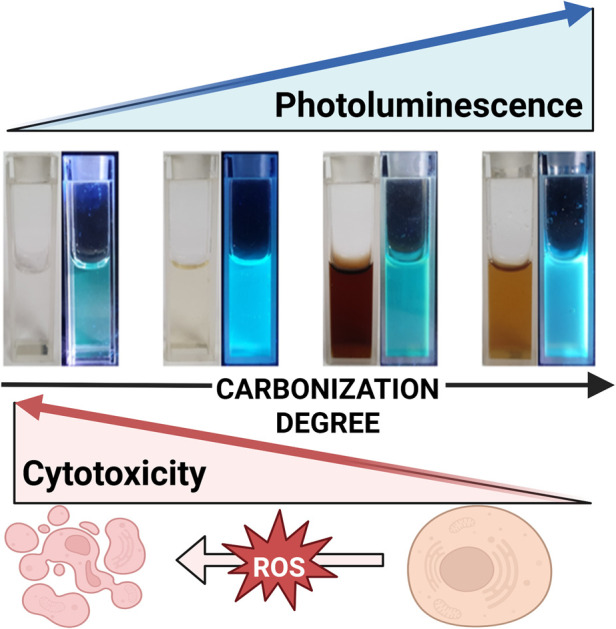

## Introduction

Nanomedicine is a field at the intersection of medicine and nanotechnology that has introduced innovations in applications such as diagnosis, imaging, and drug delivery.^1,2^ The development of nanoparticles offers advantageous properties compared to bulk materials, owing to their nanoscale-specific optoelectronic and physicochemical properties, which can be incorporated into biological systems to achieve therapeutic effects.^3,4^ Carbon dots (CDs) have gained significant attention among various types of nanoparticles. They are a class of fluorescent, zero-dimensional materials with diameters less than 10 nm, featuring a small crystalline core and polymer surface groups as functional shells.^5,6^ These nanomaterials have been reported to exhibit physicochemical properties, such as light emission, water solubility, high conductivity, and colloidal stability, along with other desirable characteristics like surface functionalization capability and biocompatibility.^7^ These properties can be modulated, enabling a wide range of applications in biomedicine, such as bioimaging, nanosensors, optoelectronic devices, and drug delivery.^8,9^

CD synthesis can be achieved through either top-down or bottom-up approaches, both of which are well described in the literature.^10^ However, particular emphasis has been placed on bottom-up methods, such as hydrothermal and microwave-assisted pyrolysis, which are among the most commonly employed. Hydrothermal synthesis is commonly used and involves a temperature-dependent polymerization and carbonization process on the surface of CDs.^11^ Nevertheless, this method has some drawbacks, such as high energy consumption, heterogeneous particle size distribution, and the presence of impurities in the final product.^12,13^ In contrast, microwave irradiation allows a more homogeneous size distribution and reduced impurity levels, though it may lead to diminished photoluminescence properties.^14,15^ Furthermore, combining these methods can enhance the desired characteristics of CDs by facilitating the formation of carbon cores in a uniform CD suspension with lower energy consumption. This approach improves structural characteristics and optoelectronic properties and influences cytotoxicity variations.^16–20^

In addition to the synthesis method, the selection of carbon source can also influence the properties of CDs. For instance, cationic carbon dots (C-CDs) exhibit a positive surface charge due to the elevated presence of amine groups, enabling fluorescent emission and targeted interactions with negatively charged cellular components, including membranes, DNA, and RNA. These interactions can potentially inhibit tumor cell growth, exert antibacterial effects, or facilitate cell permeability and adhesion, thereby enhancing therapeutic outcomes through improved cell-nanomaterial interactions.^21,22^ To address this issue, different C-CDs have been investigated for their ability to induce cytotoxicity effects on cancer cells under light exposure, acting as photosensitizing agents. However, the role of photoinduced electron transfer in C-CD-mediated cancer cell cytotoxicity remains largely unexplored.^23,24^

Furthermore, in relation to the modulation of the physicochemical properties and biological effects of CDs, it is essential to understand the two key mechanisms involved in their structural development during synthesis: polymerization and carbonization.^25^ It has been postulated that CDs more closely resemble polymer structures. Initially, they transform from polymer chains to highly crosslinked network structures (polymerization process). Then, a part of the polymer structure turns into a carbon skeleton during carbonization. These two processes usually occur uncontrollably due to the fast reaction rate and high temperature for the most used solvothermal or microwave methods.^26–29^ Thus, further studies are needed to optimize CDs' properties, particularly regarding the association between the synthesis and carbonization process, with their physicochemical properties and interaction with biological systems, where they all interplay to allow potential applications.^30,31^

To address this gap, this study aims to elucidate the intricate relationship between the polymerization and carbonization process associated with variations in heat during hydrothermal and microwave-assisted synthesis, specifically regarding the physicochemical and biological performance of C-CDs. This investigation strives to contribute valuable insights that will advance the application of carbon dots as versatile and efficient nanomaterials, with the modulation of passivation surface and the potential application in diverse areas.

## Experimental

### Chemicals

Polyethyleneimine (PEI, *M*_w_: 20 000 g mol^−1^) and chitosan (CS, *M*_w_ 190 000–310 000 Da, degree of deacetylation ≥75%, cat. no. 448 877) were purchased and used as received from Sigma-Aldrich. All the solutions were prepared using Milli Q water obtained from an Adrona CNB1901 Milli-Q Ultrapure water purification system.

### Carbon dot (CD) synthesis

CS-PEI CDs were prepared using two consecutive steps from microwave to hydrothermal synthesis, as previously established.^32^ The hydrothermal reaction was carried out at 90, 120, 150, and 180 °C to evaluate differences in the nanomaterial according to the carbonization process. Next, each procedure was described in detail.

#### Microwave synthesis step

CS-PEI CDs were prepared by adding 10 mL of 2% (m/v) of the CS sample (dissolved in 0.5 M acetic acid) to a round-bottom flask and 50 mg of PEI dissolved in 3 mL of Milli Q water. Then, the solution was microwave irradiated utilizing a domestic microwave, setting up the equipment at half-power and intervals of 0.5 min for 8 min. The time intervals were required to cool down the CD's solution. The initial and final solutions (after 8 min irradiation) were clear and transparent.

#### Hydrothermal synthesis step

Microwave-prepared CDs were further employed in the hydrothermal synthesis step. The CD solution was introduced in a polytetrafluoroethylene (PTFE) container, put in a hydrothermal reactor and sealed. Then, the system was heated at 90 °C, 120 °C, 150 °C, or 180 °C for 4 h, depending on the carbonization temperature evaluated. Afterwards, the reactor was cooled down at room temperature and the sample was recovered in a glass vial (except for CDs-180 °C, whose supernatant of the material was recovered). The hydrothermal reaction produces a transparent CD solution at 90 °C, a yellow color at 120 °C, and a black coffee color at 150 and 180 °C. All the CD formulations were centrifuged at 10 000 rpm, filtered employing a 0.22 μm filter, and stored at room temperature and in darkness.

### Characterization

The size and morphology of the CDs were recorded using a Talos F200C G2 transmission electron microscope (TEM) (Thermo Fisher Scientific) at 120 kV. The grids were prepared by adding 10 μL of CD sample suspensions on an ultrathin carbon film supported on a copper grid (400 mesh, Ted Pella, Inc.) previously treated with UV light. Furthermore, *ζ*-potential was measured by laser Doppler anemometry using a Zetasizer Nano ZS (Malvern Instruments, UK).

Molecular structure analysis of CDs was performed by evaluating the Fourier transform infrared (FT-IR) spectra using a UATR Two instrument from PerkinElmer (64 scans per sample). The samples in their powder form were obtained by lyophilizing the CDs samples using a BK-FD10 freeze-dryer (Biobase). Thermogravimetric analysis of the carbon dots was performed using a Simultaneous Thermal Analyzer (STA) 8000 (PerkinElmer). A nitrogen flux obtained the record of TGA curves at 20 mL min^−1^, and the sample was heated from 25 °C to 1000 °C at a heating rate of 10 °C per minute.

Absorption bands of CS-PEI CDs were measured in a UV-vis spectrophotometer (Jasco V-750). Fluorescence excitation and emission spectra were recorded in a Jasco FP-8300 spectrofluorometer. The excitation spectra were measured using the slit_ex_ and slit_em_ at 20 nm, while slit_ex_ and slit_em_ were 5 nm for the emission spectra. Fluorescence intensity and phase modulation profile for lifetime determination were measured in a Chronos DFD fluorescence spectrophotometer (ISS) using an excitation wavelength of 405 nm. The nanoparticle suspension fluorescence was evaluated by standard optics of 90°. Meanwhile, the fluorescence lifetime was performed using a 430 nm emission band filter to remove the excitation wavelength scattering. The results were analyzed using Vinci 3.0 Software.

### Antibacterial activity

Gram-positive *Staphylococcus aureus* (ATCC 25923) and Gram-negative *Escherichia coli* (ML-35) were employed as bacterial models for minimal inhibitory concentration (MIC) assessments following previously reported methodologies.^33^ Both strains were cultivated in Mueller–Hinton II (M–H II) medium and incubated at 37 °C overnight. Then, an aliquot was prepared by the dilution factor of 1/50 of the M–H II and incubated at 200 rpm until the bacterial culture reached an OD_600_ between 0.3 and 0.6, corresponding to the exponential phase. Afterward, the cells were centrifuged at 6000 rpm for 2 minutes, and the pellet was washed twice using 1% M–H II in 10 mM phosphate-buffered saline (PBS) as a buffer solution. Finally, the pellet was resuspended in the same wash solution, and the bacteria concentration was adjusted to introduce a total of 10^6^ CFU mL^−1^ (CFU: colony forming unit) and exposed to CD samples (from 7.8 μg mL^−1^ to 1 mg mL^−1^) overnight for 12 h in light or darkness conditions at 37 °C. A non-CD treatment was used as a negative control.

The minimum inhibitory concentration (MIC) was the lowest concentration of CD formulations that completely inhibited bacterial growth after incubation for 12 h at 37 °C. The test was realized in triplicate.

### Hemolytic activity

The experimental protocol was approved by the Bioethics and Biosafety Committee of the Universidad Autónoma de Chile (no. BE 05-23). Red blood cells (RBC) were freshly collected and immediately processed to assess the CD's hemolytic activity measurements, as previously described by Santos *et al.* (2023).^32^ Briefly, 1 mL of RBCs were spun at 2000×*g* for 10 minutes at 4 °C. Then, the supernatant was removed and the RBC pellet was washed thrice using 10 mM PBS. A 5% v/v erythrocyte suspension (∼6 × 10^8^ cells per mL) and CDs (10, 100, and 500 μg mL^−1^) were prepared with 10 mM PBS. Afterwards, an aliquot of 65 μL of erythrocyte suspension and 65 μL of CD suspensions were mixed and incubated at 37 °C for 1 h. Posteriorly, the samples were centrifuged at 3000×*g* for 5 minutes. Finally, 80-μL aliquots were added into a 96-well cell culture TPP to determine the absorbance at 540 nm in a VERSA max microplate reader. Triton X-100 at 0.5% v/v was used as a positive control of 100% hemolysis (C+), and 10 mM PBS solution was used as a negative control (C−).

The test was realized in triplicate, and the determination of hemolytic activity was calculated using the following equation:Hemolysis % = (Abs CDs − Abs C−)/(Abs C+ − Abs C−) × 100

### CD cytotoxicity assays

AGS cells (gastric cancer cell line), HeLa cells (cervical cancer cell line), HT-29 cells (colon cancer cell line), PC-3 (prostate cancer cell line), MCF-7 (breast cancer cell line) and MCF-10 (human breast epithelial cell line) were obtained from the American Type Culture Collection (ATCC, Rockville, MD, USA). All tested cell lines were maintained in a 1 : 1 mixture of Dulbecco's modified Eagle's medium (DMEM) and Ham's F12 medium containing 10% heat-inactivated fetal bovine serum (FBS), penicillin (100 U mL^−1^), and streptomycin (100 μg mL^−1^) in a humidified atmosphere with 5% CO_2_ at 37 °C.

Stock cells (AGS, HeLa, HT-29, PC3, MCF-7, and MCF-10) were incubated at 37 °C under a humidified atmosphere with 5% CO_2_ for 24 h before the assay. The starting suspension contained 3000 cells per well in a 96-well microplate. The CDs were dissolved in deionized water and diluted with the growth medium to the desired concentrations (0–500 μg mL^−1^). All culture microplates were incubated at 37 °C in a CO_2_ incubator with humidified 5% CO_2_ for 72 h. At the end of the CD exposure, cells were fixed with 50% trichloroacetic acid at 4 °C for 1 h. After washing with deionized water, cells were stained with 0.1% SRB dissolved in 1% acetic acid (50 μL per well) for 30 min and subsequently washed with 1% acetic acid to remove the unbound stain. Protein-bound stain was solubilized with 100 μL of 10 mM unbuffered Tris base, and the cell density was determined using an ELISA spectrometer plate reader at an emission wavelength of 540 nm using the Gen5 1.07 program. The data obtained were expressed as percentages of viable cells *vs.* solvent control, whose viability was considered 100%. Values shown are the mean ± S.D. of three independent experiments in triplicate. SigmaPlot® version 11.0 was used to calculate the IC_50_ values.

### Determination of the reactive oxygen species (ROS) production

Briefly, cells were treated with CD-90 (IC_50_, IC_75,_ and IC_90_, μg mL^−1^) for 12 h. Untreated cells were used as assay control, while cells treated with 4 mM of 2,2′-azobis(2-methylpropionamide)-dihydrochloride (AAPH) were used as the positive control (C+). Intracellular ROS levels were visualized after incubation with 2′,7′-dichlorodihydro-fluorescein diacetate (DCFH2-DA) at a final concentration of 10 μM. The fluorescent dye was added during the last 30 min of the extract treatment period. After the incubation, cells were washed once in PBS, trypsinized, and centrifuged. The pellet was resuspended in PBS and examined immediately by flow cytometry.^34^

## Results and discussion

### CD synthesis

**Fig. 1:**
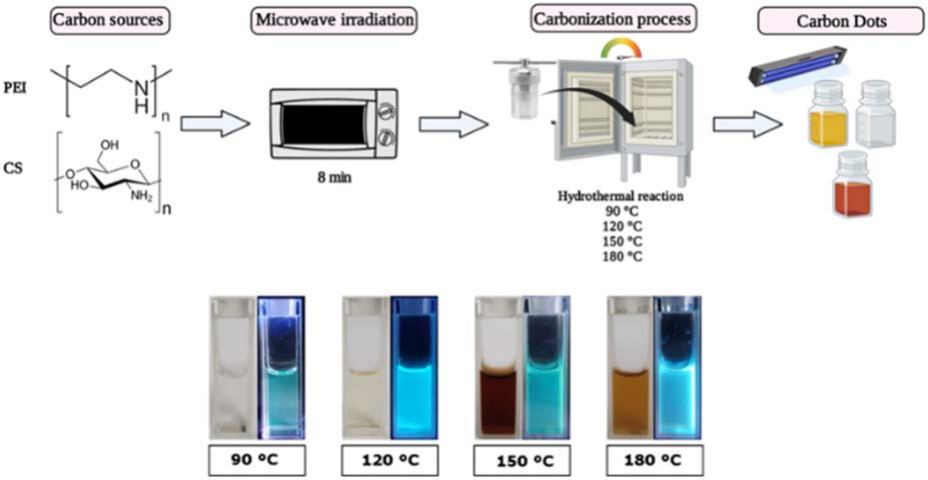
Synthesis of carbon dots (CDs) *via* microwave-assisted hydrothermal reaction at different carbonization temperatures (90, 120, 150, and 180 °C) (top). Photos of CDs when exposed to visible light (left) and under UV irradiation (365 nm; right) (bottom).

Among the cationic components, chitosan (CS) has gained significant attention as a carbon source for synthesizing CDs. This material offers several advantages, including their abundance, low cost, biocompatibility, and easy functionalization due to the presence of amine and hydroxyl groups on the surface, which improve the water solubility of the CS-derived CDs in contrast to the bulk material.^35–37^ Meanwhile, polyethyleneimine (PEI) has been commonly used in gene transfer therapy due to the permeabilization of the plasma membrane and endosomal escape of the nanoparticle by the “proton sponge” mechanism.^38^ This work used both reagents to synthesize the carbon dots (CDs) following a bottom-up approach employing a microwave-assisted hydrothermal reaction (Fig. 1a). Under this work scheme, the influence of different carbonization temperatures during the hydrothermal reaction (90, 120, 150, and 180 °C) was evaluated regarding their physicochemical and some biological properties. CDs at 90 °C (CDs-90) show characteristics like those previously reported,^39^*i.e.*, correspond to a translucid clear solution and increased temperature, while performing the hydrothermal solvolysis step drastically modified the CDs' optical properties. CDs-120 shows a lighter yellow translucid color and CDs-150 is a dark brown (yet translucid) color (Fig. 1b). At the highest temperature considered in this work, CDs-180 forms a precipitate, and polymers may be produced due to aggregation. However, centrifugation results in a brownish translucid supernatant product. All the solutions showed photoluminescence (PL) upon excitation with UV light (365 nm) but with color emission differences. Previous studies employing other carbon sources have reported the effect of the carbonization temperature on the CDs' PL properties, showing similarities to those reported here. For instance, An and co-workers (2021) obtained green and blue light-emitting CDs under UV irradiation, prepared by autoclave heating at 180 °C and 230 °C, respectively, using phenylenediamine as carbon and nitrogen sources.^40^ Esfandiari and co-workers (2019) observed that the increase in temperature in the synthesis of CDs derived from citric acid followed a color gradient, going from a clear transparent to a yellow-like color and finishing with a dark-brown colloid suspension of nanoparticles.^41^ An acetic acid solution (0.5 M) was employed during the synthesis process to dissolve the CS. Acidic conditions contribute to the oxidizing process within solvothermal pathways;^42^ high concentrations of organic acid are required to reach conditions similar to those obtained with inorganic acids.^43^ Due to the experimental conditions established for this work, extreme oxidizing conditions are discarded; however, using low/mild acidic conditions contributes to low amounts of oxygen within the CD structure and allows synthesis processes with low to non-by-product formation.^44,45^

### CDs' physicochemical characterization

**Fig. 2:**
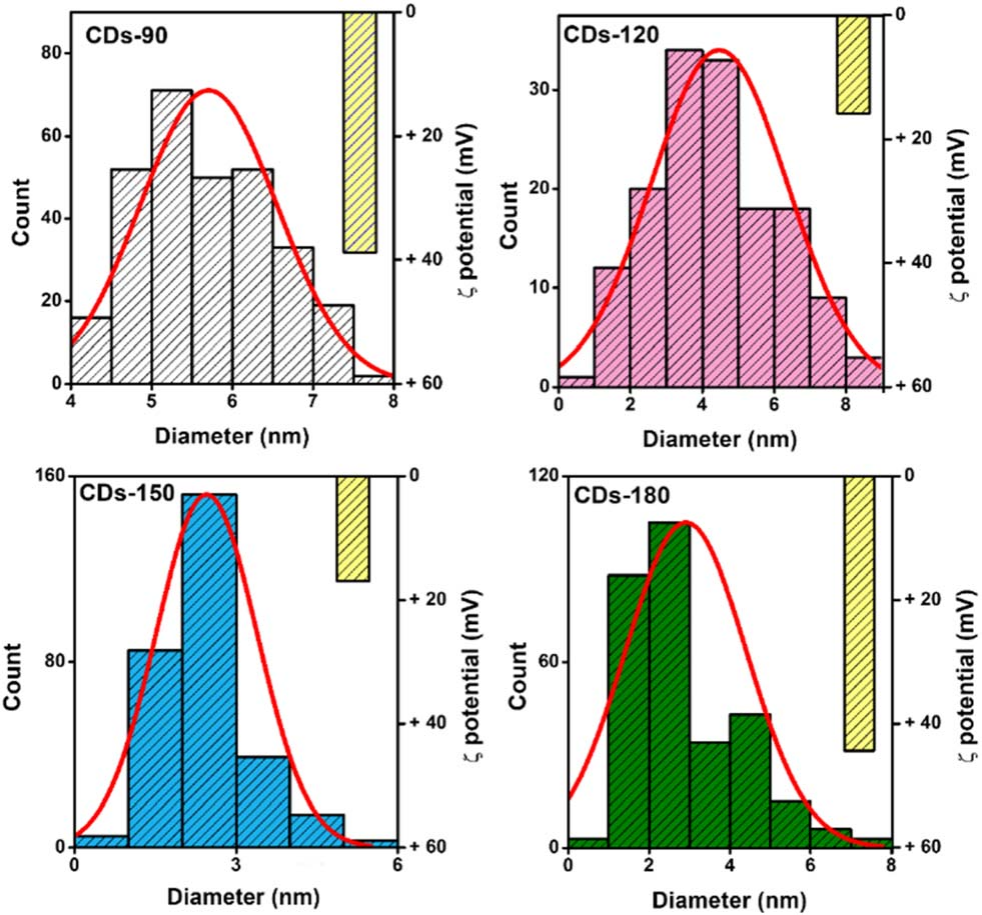
Size and measured *ζ* potential (yellow bar) of CDs. Histograms were obtained by counting 200 individual particles per sample using TEM images of the carbon dot samples.

The evaluation of CDs' morphology and size by TEM (Fig. 2 and S1†) confirmed that all synthesized samples present a quasi-spherical morphology at the different carbonization temperatures. However, differences were observed in terms of size. CDs-90 has a size of 5.7 (±0.85) nm, 4.4 (±1.89) nm for CDs-120, 2.45 (±0.94) nm for CDs-150, and 2.9 (±1.5) nm for CDs-180. While it could be considered in the first instance that smaller nanoparticles are obtained with higher temperatures, this cannot be asseverated in this work, as the size differences between all samples are non-significant, with a *p* < 0.05. Nonetheless, smaller and more condensed structures are observed at higher carbonization temperatures. Similarly to these results, Xiong and co-workers (2017) prepared CDs employing the hydrothermal method at 140 and 200 °C, utilizing ethylenediamine (EDA) as a passivation agent. They showed that the CDs have an average size of 7 nm at the highest temperature. Meanwhile, the CDs synthesized at 140 °C showed an average size of 14 nm.^46^

These results are associated with the process that follows the bulk materials when exposed to increasing temperatures.^47^ At lower temperatures, the polymers degrade, begin to polymerize, and condense, maintaining most of their functional groups in a state usually referred to as polymer dots; the temperature increase will lead to cyclization and further polymerization of the starting reagents; finally, higher temperatures promote the formation of highly complex structures, and ultimately, carbogenic cores. This compaction process over the carbon cores and the high abundance of nitrogen groups that promote an increase in the charge and repulsive force between the nanoparticles could explain the formation of smaller and condensed structures.^48^ Concurrently, Papaioannou and colleagues (2019) demonstrated the effect of the temperature on the hydrothermal reaction of CDs, which led to an increase in the carbonization process, promoting the formation of crystalline structures and CDs of smaller sizes by the reduction of the polymer shell.^49^

Due to the high presence of amine groups in the developed CDs, *ζ* potential demonstrates positive superficial charges in all nanomaterials. Nevertheless, the charge increases at the selected extreme temperatures. Carbonization processes at 90 and 180 °C produce CDs with a charge of around +40 mV, while CDs-120 and CDs-150 present a surface charge close to +15 mV. These results might suggest a variation in the composition of the elements on the CD's surface. For instance, Zhang and colleagues (2016) observed reduced oxygen content while the carbon content increased due to the carbonization process. By increasing the temperature in the citric acid/ammonia-derived carbon dots, higher N content is presented at 180 °C with a parabolic behavior in the composition of these nanomaterials due to the temperature variation in the hydrothermal synthesis process.^50^ Nevertheless, further analysis is required to attribute the surface quantification of elements to the surface charge of the carbon dots depending on the carbonization process.

**Fig. 3:**
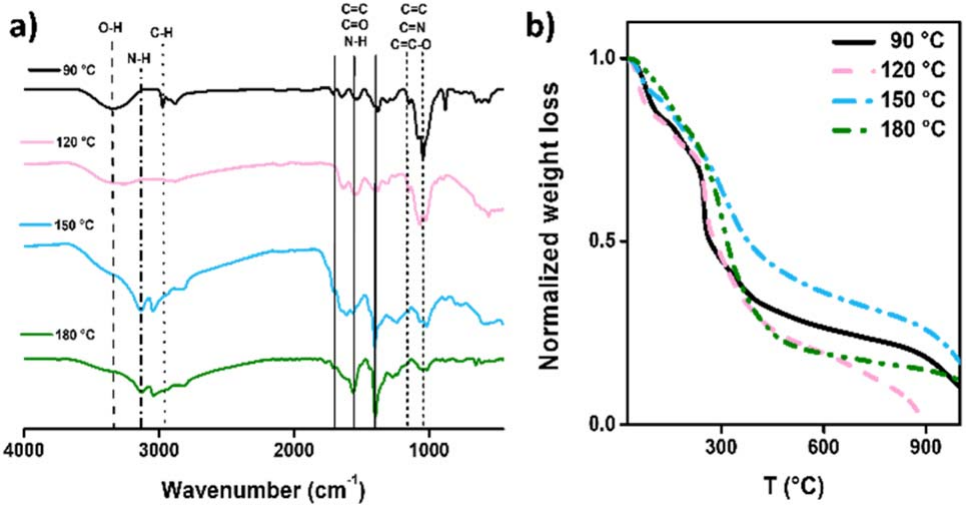
Molecular and thermal characterization of CDs: (a) FT-IR spectrum, with each spectrum consisting of 64 accumulative scans; (b) TGA thermograms obtained using a 25–1000 °C sweep and a 10 °C min^−1^ ramp.

The Fourier transform infrared (FT-IR) spectrum was measured to recognize functional groups of CDs and evaluate variation associated with the carbonization process (Fig. 3a). The characteristic functional groups of the bulk materials^32^ are presented in the CD samples at 3400, 1570, and 1410 cm^−1^ associated with the stretching vibrations of hydroxyl and amine groups for chitosan^51,52^ and amine due to the stretching vibration of C–N around 3270 cm^−1^ and 1150 cm^−1^, and the bending vibration of N–H corresponding to the PEI-based CDs at 1630 cm^−1^.^53^ The increased intensity of the associated N–H bands is a qualitative indicator that these vibrational modes intensify in the CDs as the synthesis temperature rises. As seen in Fig. 3a, the bands between 1700 cm^−1^ and 1000 cm^−1^ show a noticeable increase, consistent with the earlier discussion on the effect of temperature. Another finding is observed in the bands around 2950 and 1650 cm^−1^, which can be associated with the C–H and C=C stretching vibrations, respectively. These bands are related to the carbon sp^3^ and sp^2^ hybridizations. It is observed that with increasing temperature, the C–H band (sp^3^) tends to disappear, while the intensity of the C=C band (sp^2^) increases. Although this point requires further investigation, it suggests an increase in the sp^2^/sp^3^ ratio, indicating a rise in the crystallinity of the CDs.^54^

Additionally, CDs' thermogravimetric analysis (TGA) was performed to evaluate the thermal stability of the proposed carbonization temperatures (Fig. 3b). Compared to the base reagents (Fig. S2†), all samples presented a similar weight decline at approximately 320 °C, and a complete decrease of CDs-120 was observed at 900 °C. Meanwhile, the remaining samples presented weight stability over 1000 °C.

Concurrent with the presented results, incorporating chitosan as a carbon source in nanomaterials has several stages of thermal degradation. For instance, dehydration promoted a slight weight decline of around 195 °C. Meanwhile, the prolonged degradation until 400 °C is attributed to the decomposition of NH and OH bonds of the CS, which can continue with temperature increases over 700 °C, attributed to the organic layers of CS being removed as CO_2_.^55,56^ Furthermore, the use of PEI involves faster declines, which finish at nearly 300 °C according to the bulk PEI, and the literature corroborates that decay starts around 240 °C. Meanwhile, the first decay before 100 °C can be attributed to the nanomaterial's gases previously absorbed.^57^

### CD spectrophotometric characterization

**Fig. 4:**
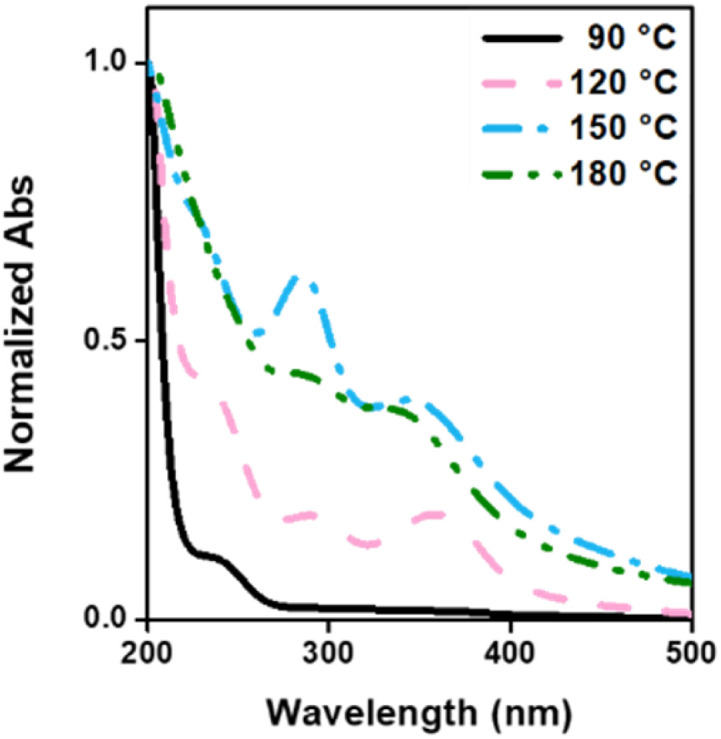
Normalized UV-vis absorption spectrum of CDs.

Under the proposed CD formulations, PEI and CS were used as carbon sources, introducing functional groups onto the surface of the carbon core. The UV-vis absorption spectrum (Fig. 4) revealed distinctive peaks associated with specific carbonization temperatures. CDs-90 and CDs-120 exhibited a characteristic peak at 250 nm, corresponding to PEI as a passivation agent. Additionally, absorption peaks around 290 and 350 nm were observed, indicating the n → π* transition of the C=O and C=N groups, respectively, which are prevalent in the carbonization process at 120, 150, and 180 °C.^58,59^ Notably, these results elucidated the appearance of an absorption peak around 290 and 350 nm, which increased with the rise in temperature until CDs-150. However, the decrease in the intensity of these peaks observed in CDs-180 suggest that over-polymerization leads to the condensation of carbon shell over the carbon core, resulting in CDs with less defined n–π* domains,^60,61^ which aligns with the previous presented results regarding the size decrease and sp^2^/sp^3^ ratio increase.

**Fig. 5:**
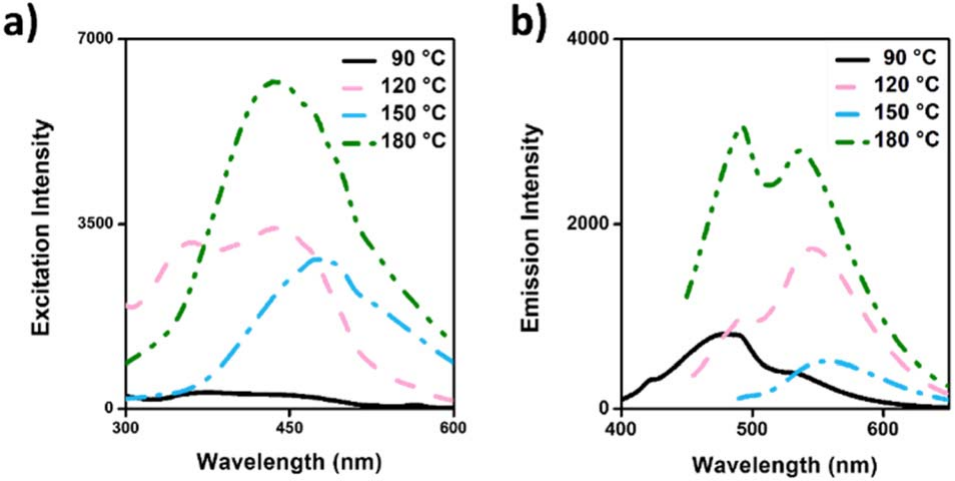
Photoluminescence properties of CD's formulations: (a) excitation spectra; (b) emission spectra.

According to the photoluminescent (PL) properties (Fig. 5), the highest excitation peak was observed in CDs-180, reflecting the carbonization process, which was also demonstrated in the FTIR results. CDs-150 and CDs-180 were evaluated at a concentration of 0.5 mg mL^−1^, while the remaining samples were assessed at 1 mg mL^−1^. The excitation-dependent emission spectra of CDs synthesized at different temperatures demonstrated a shift in excitation peaks and increased emission intensity with higher synthesis temperatures. CDs-90 showed lower excitation and emission intensities, together with the highest energy peak position found for the different formulations (480 nm), which can be expected since the polymer state (lower crystallinity) tends to predominate at low temperatures, ultimately explain this optical behavior.^62^ Further, CDs-120 showed two excitation peaks and exhibited two emission peaks upon excitation at 440 nm; considering the peak's wavelengths, this could be attributed to two CDs populations, one corresponding to the polymer dots state, while the other can be ascribed to a more carbogenic core, which aligns with previous results. CDs-150 presents a broad excitation band with an almost symmetrical emission band with a peak at 540 nm. Finally, CDs-180 showed broader and the most intense excitation and emission spectra, correlating with the sp^2^/sp^3^ ratio increase. It is expected that higher temperatures should show a similar trend behavior.^47^ These findings suggest that temperature plays a crucial role in the absorption and emission behavior of CDs, influencing the release of photons and the energy they possess.

**Fig. 6:**
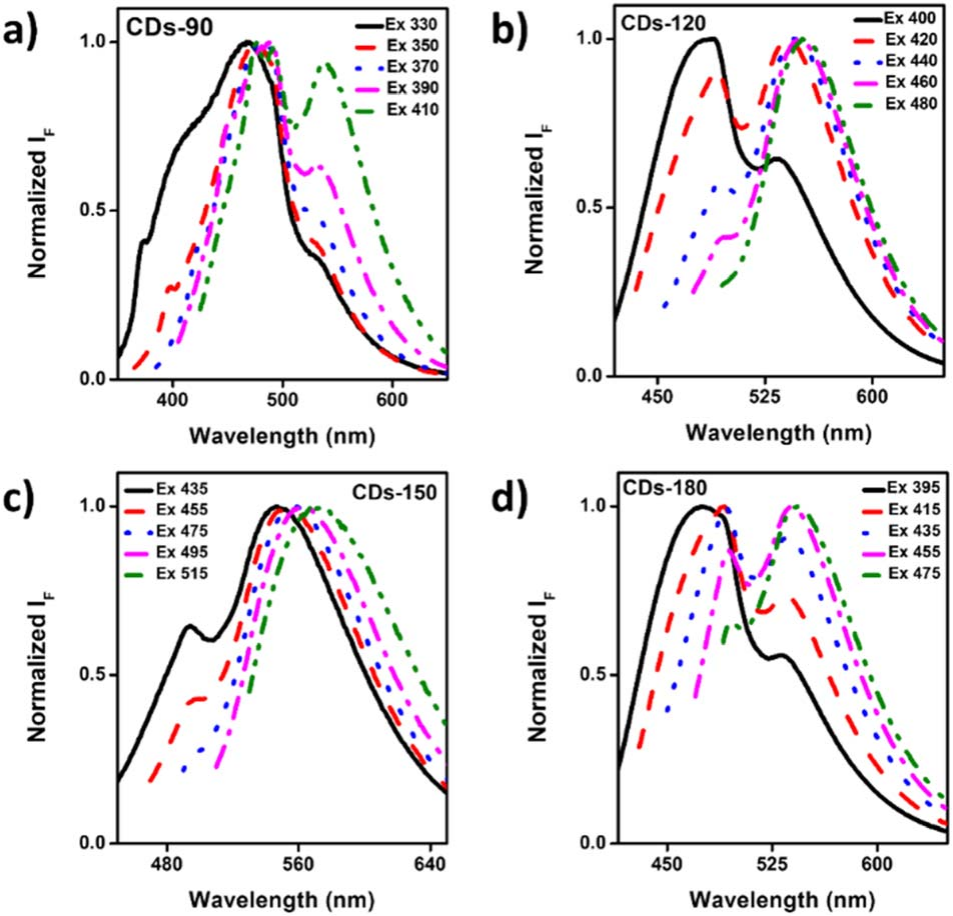
Normalized fluorescence emission spectra at various excitation wavelengths: (a) CDs-90 °C (330–410 nm); (b) CDs-120 °C (400–480 nm); (c) CDs-150 °C (435–515 nm); and (d) CDs-180 °C (395–475 nm).

According to the literature, the carbonization process induces polycondensation, forming crosslinked polymer clusters and carbonized polymer dots (CPDs). Then, the variation in the temperature during carbonization results in variation in the size and PL properties of the CDs,^48^ increasing the polymer density over the CDs core. Therefore, the conjugation between the development of the carbon core and the amine groups on the surface contributes to forming a crystalline carbon core with a highly doped nitrogen surface,^63^ which is believed to be the key factor in enhancing PL properties.^64^ Zhang and colleagues (2016) evaluated N-doped CDs designed at six temperatures (from 120 to 220 °C) and proposed the mechanism of CD formation from polymer-like NCDs to carbogenic NCDs led by increasing temperature, which promotes the growth of fluorescent polymer chains (FPC) over the CDs surface until the highest temperatures (carbogenic), observing a parabolic behavior in the PL intensity due to reduced FPC at temperatures above 180 °C.^50^ Regarding the PL behavior, the fluorescence emission at different excitation wavelengths was followed by a continuous red-shift (Fig. 6a–d), where the first emission peak was around 480 nm and shifted to nearly 530 nm. However, the trend of tunability of the CDs at different temperatures shows variation in the excitation wavelength required to change the maximum peak, which can be followed in CDs-90 (Fig. 6a) and CDs-180 (Fig. 6d). Meanwhile, the maximum peak of CDs-120 (Fig. 6b) presents a displacement to 530 nm after increasing the excitation wavelength. In the meantime, the PL behavior of CDs-150 around the emission peak continuously shifts with the increase in wavelength to 530 nm (Fig. 6c). This tunable process is usually attributed to the change in size, distribution, and surface disorder of the CDs.^65^

Furthermore, the results associated with PL lifetime (Table S1†) showed a decay of the average lifetime over the increase in temperature in the hydrothermal process (from 6.12 to 5.44 ns). Therefore, it highlights the essential role of temperature in the design, and how CD's absorb energy through photons or other particles and then emit light after being excited by these sources, which can be explained by the reduction of the diameter size of CDs through an increase in temperature that leads to a change in the HOMO/LUMO energy levels.^25^ This phenomenon has been previously evaluated in CDs where the temperature rise is associated with a PL lifetime decay related to the reduction of polymer functional groups.^66^

These findings are consistent with previous studies, as tri-exponential decay was observed for all tested formulations, indicating the presence of different emissive species corresponding to fluorophores or energies aligned with earlier data sets. Thus, a better understanding of the excitation mechanisms within these systems will lead us toward more effective material design strategies by exploiting their unique optical properties, such as absorption/emission spectra. This will offer new possibilities for application fields like displays, lighting sources, or bioimaging tools, among others.^67,68^

### Antibacterial assay

The minimum inhibitory concentration (MIC) assay was conducted to evaluate the antibacterial activity of CDs synthesized through carbonization. The MIC values were determined for *S. aureus* and *E. coli* to assess the impact of carbonization temperatures on Gram-positive and Gram-negative bacteria, respectively (Table 1). Only CDs-90 exhibited an antibacterial effect among the CDs samples. The MIC for *E. coli* and *S. aureus* was 125 μg mL^−1^ and 31.2 μg mL^−1^, respectively in the presence of light. In contrast, the other CDs samples did not demonstrate an antibacterial effect, even at 1 mg mL^−1^.

**Table 1:** Minimum inhibitory concentration (MIC) of carbon dots after 24 h under light or dark conditions

Sample	*E. coli*		*S. aureus*	
	Light (μg mL^−1^)	Darkness (μg mL^−1^)	Light (μg mL^−1^)	Darkness (μg mL^−1^)
CDs-90	125	>1000	31.2	>1000
CDs-120	>1000	>1000	>1000	>1000
CDs-150	>1000	>1000	>1000	>1000
CDs-180	>1000	>1000	>1000	>1000

CDs-90 displayed a light-dependent antibacterial effect, suggesting a photoexcited process. CDs can act as photosensitizer agents, promoting the increase of reactive oxygen species (ROS) during the excitation of the nanoparticle; the electron from the conductive band spin flips to generate a triplet excited state, which reacts with an oxygen molecule to form singlet oxygen that can finally damage multiple cellular components, showing a potential promise as a nanomaterial for photodynamic treatment in bacterial infections.^69,70^

Related to the antibacterial effect, Zhao and colleagues (2022) evaluated CDs derived from chitosan quaternary ammonium salt (QCS) and ethylenediamine (EDA) as carbon sources, synthesizing them using a one-step hydrothermal method at 200 °C for 4 h. The nanomaterial design shows a significant antibacterial efficacy at 10 μg mL^−1^ against *S. aureus* and 50 μg mL^−1^ for *E. coli*, demonstrating the high efficiency of these CDs in exerting antibacterial effects with remarkable activity toward Gram-positive bacteria.^71^ The antibacterial activity was attributed to membrane cell disruption promoted by the CDs' high positive surface charge.^72,73^ Meanwhile, the small size of the nanomaterial allows it to penetrate the bacteria,^74^ promoting a loss of the DNA's double helix structure by the CDs-DNA interaction.^75^ Furthermore, the light activation of the antibacterial activity promoted by the photosensitizer-like behavior of CDs can generate reactive oxygen species (ROS) with ^1^O_2_ or free radicals, causing the death of bacteria and tissue damage by a determinate excitation wavelength.^71^ On the other hand, CDs-PEI exhibits the highest cytotoxicity with a MIC value of 5 μg mL^−1^ without light exposition due to the transfection properties of PEI, as observed in Havrdova and co-workers' (2016) research.^38^

The surface passivation molecules of PEI and CS are presented as polymer chains with antibacterial effects in CDs-90. However, the antibacterial effect was entirely diminished as the carbonization temperature increased. These results might be associated with developing carbon cores through temperature increases. In this regard, the lowest temperature employed (CDs-90) supports the idea of the obtaining a condensed structure. In contrast, the reaction might promote the formation of polymer-like CDs that produce the antibacterial effect compared to the higher temperatures, which may lead to the development of carbon cores with reduced short fluorescent polymer chains.^50^

### Carbonization temperature and hemolytic activity

**Fig. 7:**
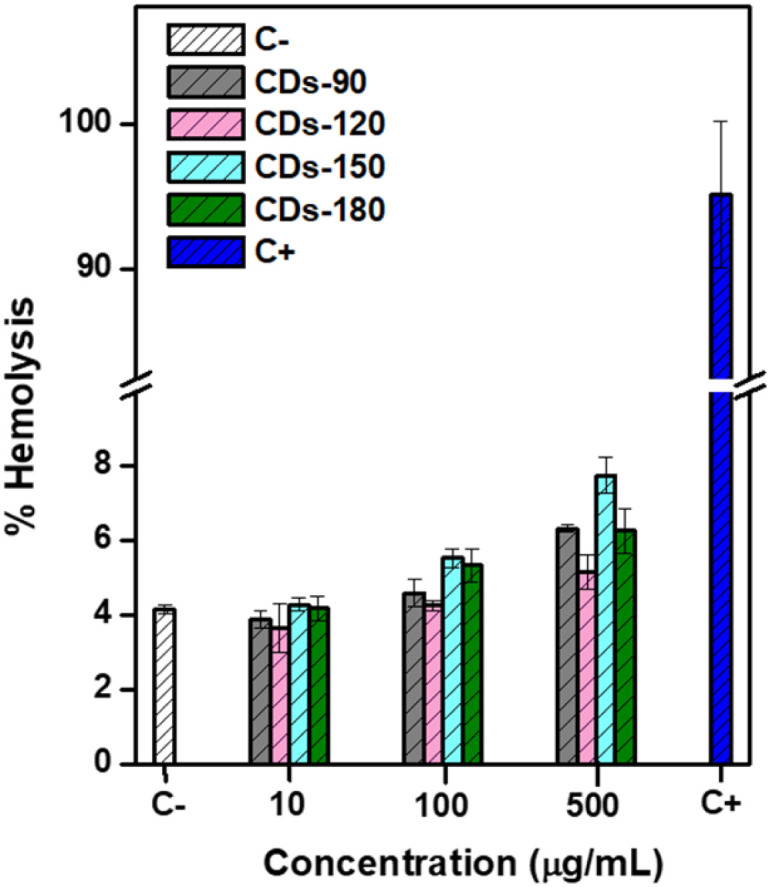
Hemolytic activity of CDs. Hemolysis (%) of mouse red blood cells (RBCs) incubated with CDs (synthesized at different temperatures) at 37 °C for 1 h.

The hemolysis assay assessed the potential toxicity and cell membrane damage associated with the carbonization effect through hydrothermal synthesis. The positive control with 0.5% Triton X-100 exhibited complete disruption of erythrocytes (Fig. 7), resulting in a red solution indicative of 100% hemolysis. In contrast, the negative control with 1× PBS pH 7.4 showed no hemolysis activity.

To evaluate the biocompatibility of the different CDs samples, erythrocytes were treated with varying concentrations of CDs (10, 100, 500 μg mL^−1^). Overall, the evaluated CDs were hemocompatible. The results are consistent with previous research that assessed the blood biocompatibility of CDs. For instance, Zhong *et al.* evaluated N-doped CDs synthesized *via* a solvothermal method and reported hemolysis below 0.5% at 300 μg mL^−1^.^76^ Similarly, microwave-assisted synthesis of CDs showed good biocompatibility, with a hemolysis rate close to 6% at 250 μg mL^−1^.^77^ These studies utilized different surface conjugation techniques, such as histidine and cysteine. In a previous work by Santos *et al.* (2023), CDs with different formulations (employing PEI and CS) and synthesized *via* microwave pyrolysis, hydrothermal microwave-assisted synthesis, and a combination of both methods, demonstrated a higher hemolysis percentage when carbonized at 90 °C, compared to CDs formulated using only the microwave method.^32^ Nevertheless, it was not possible to find more studies associated with PEI-CS-derived carbon dots.

The results indicate that the carbonization process of PEI-CS-derived CDs exhibited low cytotoxicity, even at the highest concentrations evaluated (500 μg mL^−1^). CDs-120 showed the lowest hemolytic activity, indicating the best biocompatibility among the tested samples. Furthermore, CDs-150 and CDs-180 displayed a reduced hemolysis rate compared to CDs-90 at a concentration of 500 μg mL^−1^, suggesting their potential to minimize cytotoxic effects. Overall, these hemolysis assay results support the favorable biocompatibility of the CDs developed, highlighting their potential applications in various biomedical fields. However, further investigations should be conducted to explore the underlying mechanism of biocompatibility and cytotoxicity and to assess their performance in specific biomedical applications.

### CDs' cytotoxic effect on tumoral and non-tumoral cell lines

The cytotoxicity of the CDs was evaluated by a colorimetric sulphorhodamine B assay in six different human cell lines: gastric adenocarcinoma (AGS), cervical cancer (HeLa), colon cancer (HT-29), breast cancer (MCF-7), prostate cancer (PC-3), and breast epithelial (MCF-10). According to the IC_50_ value, all CDs except CDs-90 showed a non-cytotoxic effect under 500 μg mL^−1^ in different human cell lines analyzed. Only CDs-90 for AGS, HeLa, PC-3, and MCF-10 cell lines showed IC_50_ values lower than 500 μg mL^−1^ (Table 2). Considering the carbon sources, previously developed CDs have demonstrated a reduction of the cytotoxic effect compared to the bulk PEI (as a starter reagent), as presented by Bu and co-workers (2020).^78^ A similar effect of cytotoxicity reduction was observed by Fan and co-workers (2019), where CDs were developed using the hydrothermal method.^79^ Furthermore, Havrdova and colleagues (2016) evaluated the functionalization of CDs' surface with PEI polymer, which has enhanced cytotoxicity, obtaining an IC_50_ of around 50 μg mL^−1^ in NIH/3T3 fibroblasts.^38^ The results presented here show a reduction in the cytotoxic effect of the designed CDs with increasing temperature, attributed to the development of the carbon core and carbonization of the polymer shell from the PEI passivation agent. This process may lead to decreased cell viability, similar to the observations by Han and Na (2019), where microwave-assisted pyrolysis of PEI-derived CDs enhanced biocompatibility compared to the raw material, as seen in cytotoxicity and transfection tests with hMSCs.^80^

**Table 2:** IC_50_ values of cytotoxic activity of carbon dots in various cell lines

IC_50_ μg mL^−1^	Cell line					
	AGS	HeLa	PC-3	HT-29	MCF-7	MCF-10
CDs-90	485.3 ± 34.2	276.3 ± 24.9	273.7 ± 31	>500	>500	213.6 ± 26.5
CDs-120	>500	>500	>500	>500	>500	>500
CDs-150	>500	>500	>500	>500	>500	>500
CDs-180	>500	>500	>500	>500	>500	>500

A low cytotoxic effect of the CDs was observed at 120, 150, and 180 °C. Previous studies obtained similar results. For instance, Sachdev and co-workers (2014) evaluated CS-PEI CDs synthesized hydrothermally at 200 °C. The designed material did not present a cytotoxicity effect in A549 and BHK-21 cells even at 1 mg mL^−1^, showing a potential use for bioimaging applications.^52^ As observed by Jiang and co-workers (2022), the hydrothermal synthesis of CDs derived from citric acid and PEI at 180 °C for 6 h resulted in CDs that were evaluated in a cytotoxicity test using human UC-MSCs and showed a cell viability of around 80% after 48 h of treatment with CDs at 800 μg mL^−1^.^81^ Furthermore, in another study, Esfandiari and colleagues (2019) obtained CDs from 160 to 220 °C *via* the carbonization process and evaluated their potential cytotoxicity in breast cancer SKBR3 and normal MCF-12A cells using an MTT assay. They demonstrated that the tested CDs did not exhibit cytotoxicity effects at concentrations of 1 mg mL^−1^. Nevertheless, at the highest concentration of the lowest temperature treatment (160 °C), the CDs showed a low cytotoxicity effect compared to those treated at higher temperatures, which displayed good biocompatibility, with more than 90% cell viability at 1.5 mg mL^−1^. However, the results of this work present an increase in the PL effect of CDs with the rise in temperature, showing a reduction in cytotoxicity. This contrasts with the CDs reported in the literature, where the PL properties of CDs derived from citric acid decreased at higher temperatures.^41^

Our findings indicate that CDs derived from PEI and CS exhibited a varied cytotoxic profile across different cell lines. Notably, CDs-90 demonstrated a cytotoxic effect in selected cell lines. The cytotoxic profile of the CDs-90 was evaluated *via* intracellular ROS production, where MCF-10 was treated with dichloro-dihydro-fluorescein diacetate (DCFH_2_-DA) as a quantitative measure of general oxidative stress (Fig. S3a†). Our results displayed a strong fluorescence signal of DCF-DA with MCF-10 treated with the different concentrations of CDs-90 after 12 h. The fluorescence intensity between the IC_50_ and the positive control did not present a significant difference. Meanwhile, higher concentrations, IC_75_ and IC_90_, demonstrate even more marked increases, reaching significant ROS levels higher than the cells treated at IC_50_ and the positive control. This pattern suggests a dose–response relationship in ROS generation induced by CDs-90. This effect is observed in complementary visualization through DCF fluorescence analysis of the treated cells by distribution curves (Fig. S3b†). More cells exhibit elevated levels of oxidative stress related to the intracellular ROS with increasing CDs concentration, evidenced by higher fluorescence intensity.

Our results are consistent with previous research by Ding and colleagues (2021) showing an increase in intracellular ROS in UM cells treated with CDs derived from citrate and l-tryptophan. The authors attributed this effect to the carboxy and hydroxy groups presented on the CDs' surface, leading to intracellular ROS accumulation.^82^ Furthermore, a CD-dose-dependent cytotoxicity effect was observed by Zhang and co-workers (2020), where the higher concentration at 400 μg mL^−1^ represents a significant increase in intracellular ROS. Besides, the results showed double-membrane-based vacuole formation in Hepa1-6 cells treated with CDs.^83^ Similarly, our results show various stages of degradation in MCF-10 cells treated with CDs at 225 and 500 μg mL^−1^ (Fig. S4†).

## Conclusions

This study synthesized cationic carbon dots derived from chitosan and polyethyleneimine through the microwave-assisted hydrothermal method. The presented nanomaterials showed increased photoluminescence intensity with greater temperatures during the carbonization process in the hydrothermal step. Meanwhile, there is a reduction in the cytotoxicity of mammalian cells and antibacterial effects. CDs-90 showed a minimum inhibitory concentration of 31.2 and 125 μg mL^−1^ in *S. aureus* and *E. coli*, respectively under laboratory light conditions. Furthermore, CDs-90 showed an IC_50_ lower than 500 μg mL^−1^ in four different cell lines: AGS, HeLa, PC-3, and MCF-10. Besides, general oxidative stress was evaluated in MCF-10 cells, revealing a CD90 dose-dependent increase in intracellular ROS levels. Therefore, the obtained results indicate a clear dependency of CDs' physicochemical and biological properties with the synthesis temperature, with higher temperatures allowing smaller sizes, higher photoluminescence, and lower cytotoxicity toward bacteria and mammalian cell lines, while CDs prepared at lower temperatures can promote oxidative stress. In summary, while further research should be performed to explore other formulations and experimental conditions, this study provides valuable insight into the design of CDs with the implication of the synthesis process to modulate their physicochemical properties and biological performance.

## Data availability

The article and the ESI contain the data supporting this study.†

## Author contributions

NS conducted the comprehensive investigation (experimental section) and contributed to the formal analysis and manuscript writing. PAS conducted the investigation of the hemolytic and antibacterial effects of the CDs and contributed to manuscript writing. IOR contributed to the fluorescence and lifetime experiments and assisted with manuscript writing. CJG and JV designed and performed the *in vitro* cytotoxicity experiments on the various cancer cell lines, and contributed to data analysis and manuscript writing. MA was responsible for conceptualization, formal analysis, methodology, resource acquisition, supervision, and overall manuscript writing.

## Conflicts of interest

There are no conflicts of interest to declare.

## Supplementary Material

RA-015-D5RA00062A-s001
