# Supplementary material for: Effect of temperature on the carbonization process of cationic carbon dots: a physicochemical and in vitro study
Source: RSC Adv. 2025 Apr 28;15(16):12814–24. doi: 10.1039/d5ra00062a (PMC12035528; doi:10.1039/d5ra00062a)
Supplement: RA-015-D5RA00062A-s001 [file RA-015-D5RA00062A-s001.pdf]

Supplementary Information (SI) for RSC Advances.
This journal is © The Royal Society of Chemistry 2025

Supplementary Information

Temperature effect over carbonization process of cationic carbon dots: a physicochemical and *in vitro* study

Nicolás Santos,[a] Paula A. Santana,[b] Igor Osorio-Roman,[c] Carlos Jara-Gutiérrez,[d] Joan Villena,[d] Manuel Ahumada[a,e,*]

[a]Centro de Nanotecnología Aplicada, Facultad de Ciencias, Ingeniería y Tecnología, Universidad Mayor, Camino La Pirámide 5750, Huechuraba, Santiago, RM, Chile.

[b]Instituto de Ciencias Aplicadas, Facultad de Ingeniería, Universidad Autónoma de Chile, el Llano Subercaseaux 2801, San Miguel, Santiago, Chile.

[c]Instituto de Ciencias Químicas, Facultad de Ciencias, Universidad Austral de Chile, Isla Teja s/n, Valdivia, Región de los Ríos, Chile.

[d]Centro interdisciplinario de investigación biomédica e ingeniería para la salud (MEDING), Escuela de Medicina, Facultad de Medicina, Universidad de Valparaíso, Valparaíso, Chile.

[e]Escuela de Biotecnología, Facultad de Ciencias, ingeniería y Tecnología, Universidad Mayor, Camino La Pirámide 5750, Huechuraba, Santiago, RM, Chile.

Corresponding author´s email: Manuel.ahumada@umayor.cl

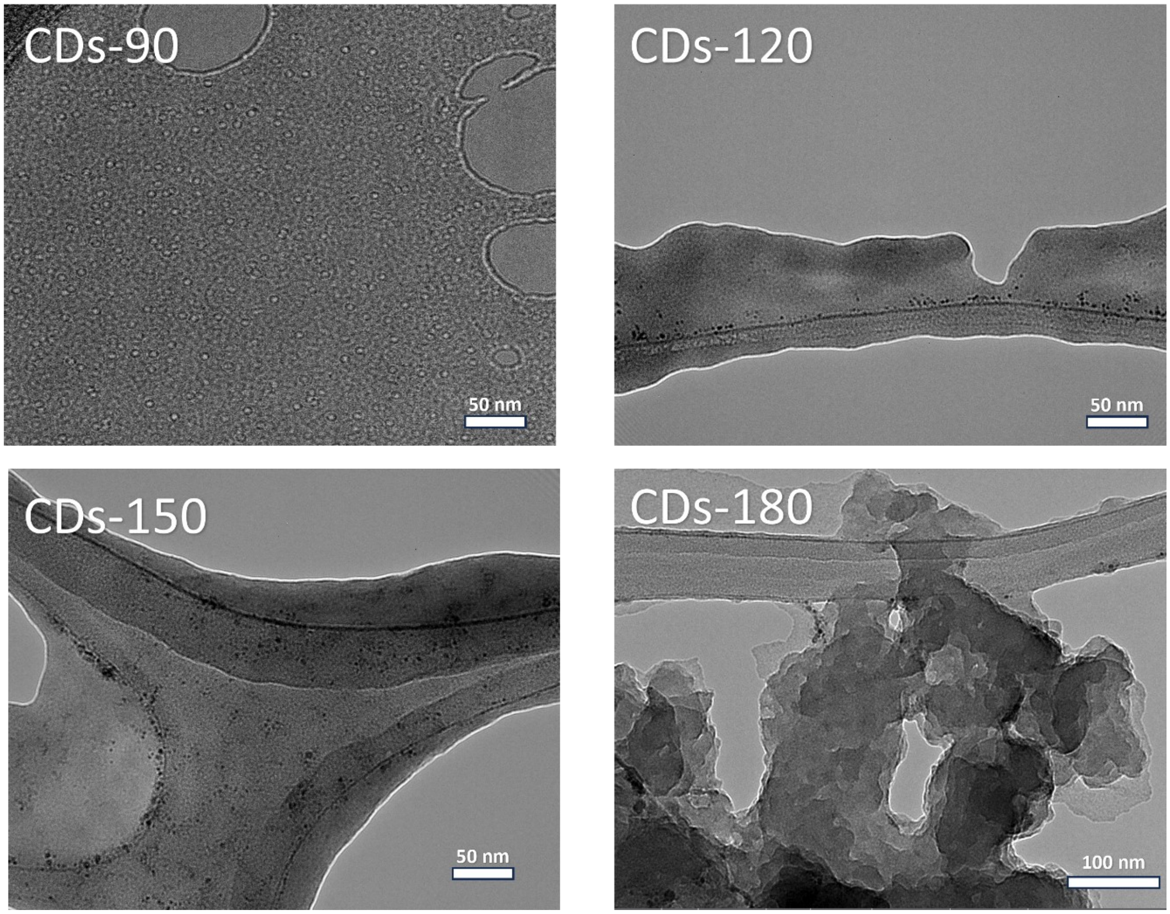

Figure S1. Representative TEM images of CDs´ formulations at different synthesis temperature.

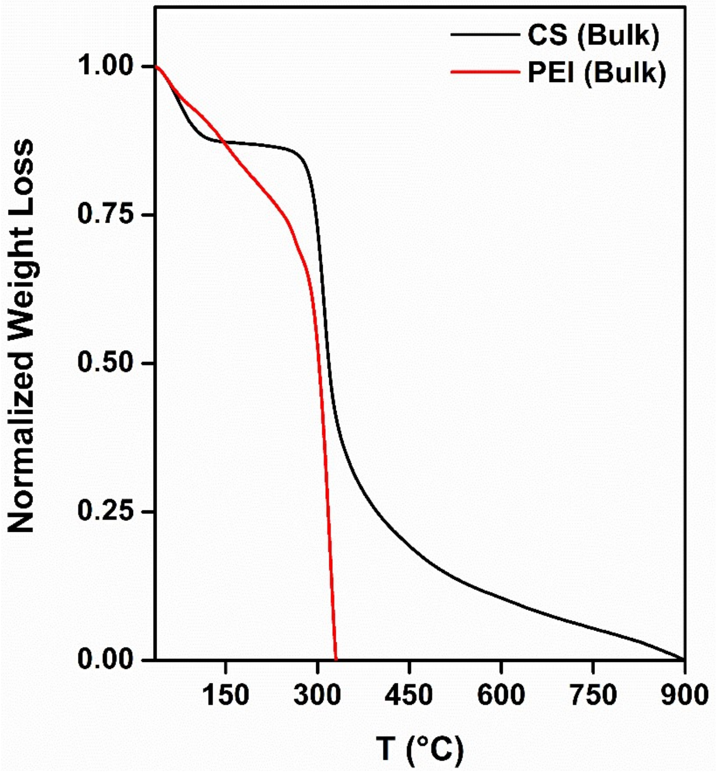

Figure S2. TGA thermograms of starting bulk reagents. Chitosan (CS; black line) and poly(ethylene imine) (PEI; red line).

Table S1. Lifetime measures of Carbon dots samples.

Sample	τ 1 (ns)	τ 2 (ns)	τ 3 (ns)	Average Lifetime (ns)	χ 2
90 ºC	3.25	0.515	10.7	6.12	2.1
120 ºC	3.2	0.515	10.1	5.95	2.18
150 ºC	2.97	0.45	9.81	5.47	1.56
180 ºC	3.03	0.493	9.52	5.44	1.18

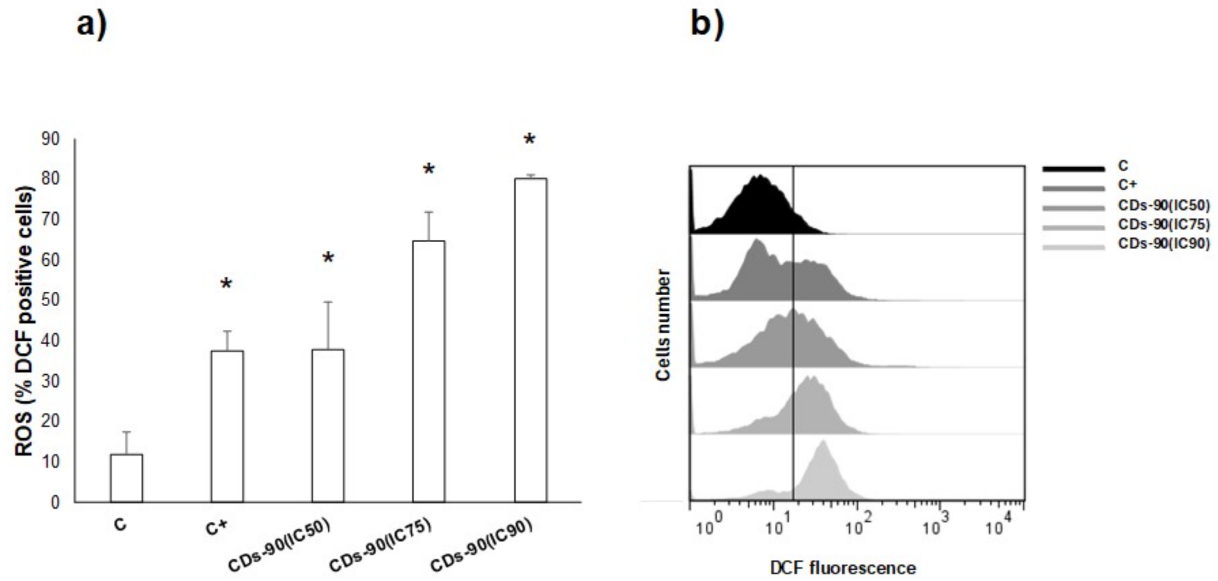

Figure S3. Effect of the CDs-90 on ROS production. Cells were treated with CDs (IC_{50}-IC_{75}-IC_{90} for 12 h), and 2,2′-azobis (2-methylpropionamide)-dihydrochloride (AAPH) 4 mM as the positive control (C+). Intracellular ROS levels were determined in the breast epithelial cell line, MCF-10, by flow cytometry using dichloro-dihydro-fluorescein diacetate ($DCFH_2$-DA). a) Histograms quantifying the detailed graph in b). * $p < 0.05$ versus control-treated cells (C). The data represent the means ± S.D. of at least three experiments with triplicate samples.

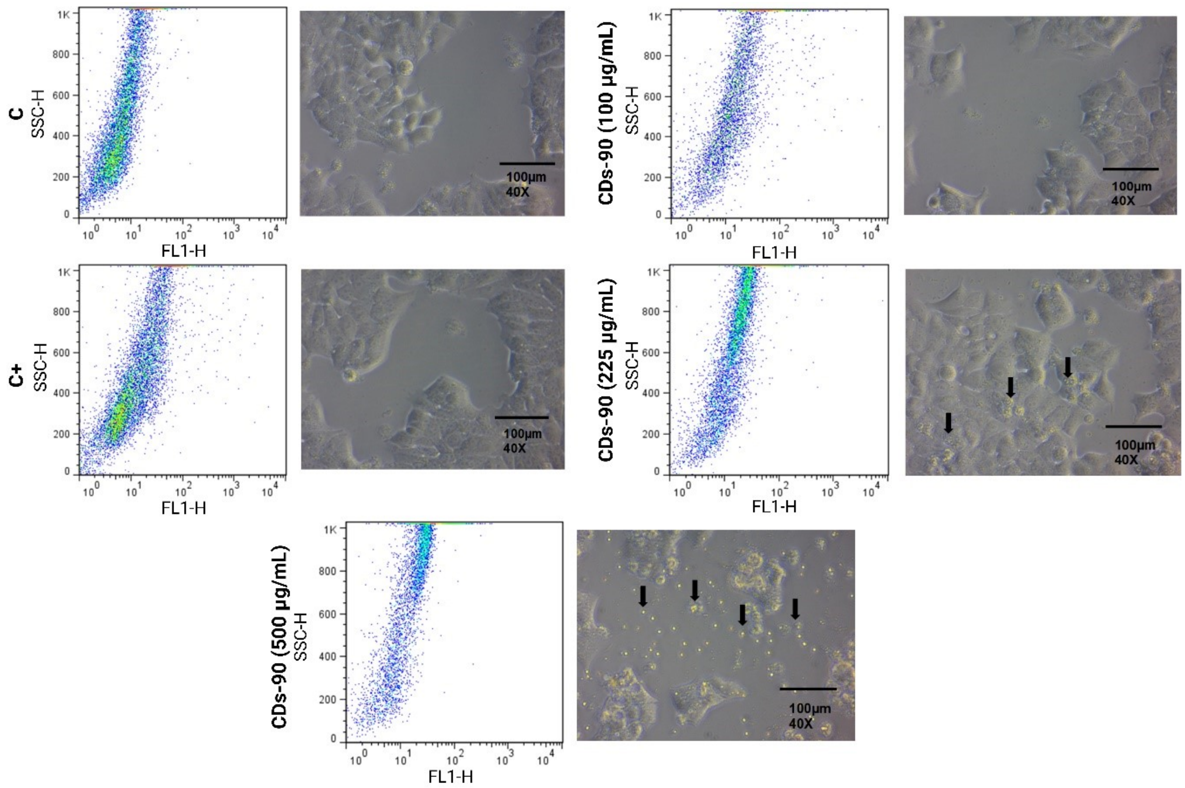

Figure S4. MCF-10 cells have various degradation stages when exposed to CDs-90 at concentrations equal to the IC50, IC75, and IC90. Further, AAPH was used as positive control (C+), and cell without anything as negative control (C). Left, density plot obtained through flow cytometry; and, Right, bright-field microscopy images, scale bars correspond to 100 µm.
